# A genome-wide detection of selection signatures in conserved and commercial pig breeds maintained in Poland

**DOI:** 10.1186/s12863-018-0681-0

**Published:** 2018-10-22

**Authors:** Artur Gurgul, Igor Jasielczuk, Katarzyna Ropka-Molik, Ewelina Semik-Gurgul, Klaudia Pawlina-Tyszko, Tomasz Szmatoła, Magdalena Szyndler-Nędza, Monika Bugno-Poniewierska, Tadeusz Blicharski, Karolina Szulc, Ewa Skrzypczak, Jędrzej Krupiński

**Affiliations:** 1 National Research Institute of Animal Production, Department of Animal Molecular Biology, Krakowska 1, 32-083, Balice, Poland; 2 National Research Institute of Animal Production, Department of Pig Breeding, Krakowska 1, 32-083, Balice, Poland; 3 Institute of Genetics and Animal Breeding, Department of Genomics and Biodiversity, Postępu 36A, Jastrzębiec, 05-552 Poland; 4 Poznań University of Life Sciences, Faculty of Veterinary Medicine and Animal Science, Wojska Polskiego 28, 60-637 Poznań, Poland; 5 National Research Institute of Animal Production, Department of Horse Breeding, Krakowska 1, 32-083, Balice, Poland

**Keywords:** F_ST_, Pig, Polish landrace, Puławska, REHH, Selection signatures, Złotnicka

## Abstract

**Background:**

Identification of selection signatures can provide a direct insight into the mechanism of artificial selection and allow further disclosure of the candidate genes related to the animals’ phenotypic variation. Domestication and subsequent long-time selection have resulted in extensive phenotypic changes in domestic pigs, involving a number of traits, like behavior, body composition, disease resistance, reproduction and coat color. In this study, based on genotypes obtained from PorcineSNP60 Illumina assay we attempt to detect both diversifying and within-breed selection signatures in 530 pigs belonging to four breeds: Polish Landrace, Puławska, Złotnicka White and Złotnicka Spotted, of which the last three are a subject of conservative breeding and substantially represent the native populations.

**Results:**

A two largely complementary statistical methods were used for signatures detection, including: pairwise F_ST_ and relative extended haplotype homozygosity (REHH) test. Breed-specific diversifying selection signals included several genes involved in processes connected with fertility, growth and metabolism which are potentially responsible for different phenotypes of the studied breeds. The diversifying selection signals also comprised *PPARD* gene that was previously found to have a large effect on the shape of the external ear in pigs or two genes encoding neuropeptide Y receptors (Y2 and Y5) involved in fat deposition and stress response which are important features differentiating the studied breeds. REHH statistics allowed detecting several within-breed selection signatures overlapping with genes connected with a range of functions including, among others: metabolic pathways, immune system response or implantation and development of the embryo.

**Conclusions:**

The study provides many potential candidate genes with implication for traits selected in the individual breeds and gives strong basis for further studies aiming at identification of sources of variation among the studied pig breeds.

**Electronic supplementary material:**

The online version of this article (10.1186/s12863-018-0681-0) contains supplementary material, which is available to authorized users.

## Background

Numerous studies have shown applicability of genomics in the field of quantitative genetics and identification of sources of variation of important phenotypic features such as production traits [1]. Unfortunately, research in this field in most cases requires numerous study populations, well characterized in terms of phenotype, relatedness structure and genome features. Due to the high cost of conducting such research, generated by the need to establish genotypes and phenotypes of large animal populations, attempts have been made to identify traces left in animal genomes by selection pressure directed at the consolidation or improvement of particular phenotypic traits [2]. The identification of selection signatures assumes detection of genomic regions in which gene variants subjected to a rapid increase in allele frequencies under the influence of selective pressure (ongoing selection) are located or detection of genome regions that are fixed in a population with well-established phenotypical features. Detection of selection signals in conjunction with subsequent candidate gene identification approach may indicate the location of major genes responsible for selected traits [3]. The advantage of such approach is that it is independent of the availability of detailed information on the phenotype of individual animals and is applicable to relatively small study populations [2].

Similarly, as other livestock species, pigs have been under long term selection during domestication, breeds formation and further improvement of production and functional traits. To analyze the mechanism underlying phenotypic differentiation caused by selection in pigs, the evidence of selection has been searched in genomes of various pig breeds using whole genome genotype data or high-throughput sequencing [4–9]. The studies allowed detecting several selection signals associated with growth traits, reproduction traits, coat color or ear phenotype and to indicate several genes with major effects on these traits [5, 7]. Nevertheless, selection patterns in pig breeds differ depending on their evolution and breeding histories, so exploration of selection signatures in possibly the largest number of different breeds will help to better understand the genetic variation underlying the traits of interest.

In the present study, we detected selection signatures at the whole genome level in three conserved pig breeds derived from the native pig populations (Puławska, Złotnicka White and Złotnicka Spotted) and a commercial Polish Landrace breed, differing in terms of production, reproduction characteristics and exterior features. Among the native breeds we included Puławska breed which is valued for good resistance to harsh environmental conditions and diseases, taste of meat (its aroma and juiciness) and usefulness for extensive breeding in ecological farms. Two other studied Złotnicka pig breeds (White and Spotted) are characterized by similar functional features but have worse productivity than Puławska breed and have more primitive character. Especially Złotnicka Spotted is being considered as a meat-lard type breed and is characterized by high subcutaneous fat content and relatively low fertility [10–13]. The detailed characteristic of the studied breeds is presented in Additional file 1.

To identify selection signatures in the analyzed breeds, we applied two different methods: first, based on F_ST_ (classical measure of population differentiation) [14] and aimed at identification of among-breeds diversifying selection and second, based on relative extended haplotype homozygosity (REHH) [15] statistics allowing for detection of mainly within-breed ongoing selection. Both F_ST_ and REHH statistics were shown to be useful to detect selection signatures [16] and they are largely complementary, because REHH test has good power to detect selection signatures within breeds and is more accurate in case of ongoing selection, while F_ST_ is useful to detect selection signatures across breeds, represented mainly by loci that were differentially fixed in different breeds [17].

Despite the lack of typical selection in terms of production in the case of conservative breeding, the animals’ qualification into the conservation program (based on breed standard and aimed at consolidation of breed-specific traits and stabilization of exterior) may also be considered as an extensive selection and lead to similar but less pronounced changes in the frequency of alleles. The applied combination of breeds and statistical methods allowed us to search for selection signals associated with both fixed traits of individual breeds and features that are still under improvement, which may help to better understand adaptation of breeds to local environmental conditions and help to evidence processes behind good health, longevity and low environmental requirements of native pigs.

## Methods

### Animals and genotyping

The material of the study was genomic DNA obtained from blood or hair bulbs of 530 animals sampled from each of the four pig breeds: Polish Landrace (PL, *n* = 135), Puławska (PUL; *n* = 155), Złotnicka White (ZW; *n* = 141), Złotnicka Spotted (ZS; *n* = 99) differing in terms of production, reproduction and exterior features. The animals were selected to be unrelated for at least two generations and originating from different herds. Each population sample included at least 7% of males. This was because we analyzed breeding population (reproductive studs) in which share of males is matched to the number of boars which are designed to natural matting. All animal procedures were approved by the Local Animal Care Ethics Committee No. II in Kraków - permission number 1293/2016 in accordance with EU regulations. The genomic DNA was purified using a Sherlock AX kit (A&A Biotechnology) and after quality control was genotyped with the use of the PorcineSNP60 BeadChip assay (Illumina) according to the standard Infinium Ultra protocol. The obtained genotypes were controlled for quality by evaluation of call rates and only samples with more than 97% of called genotypes were used for further analysis. Of the 61,565 assayed SNPs, a panel of 50,485 markers was further obtained by removing SNPs mapped to contigs, located on the sex chromosomes (Sscrofa10.2 genome assembly) or classified as intensity-only probes.

### Data analysis

The initially filtered SNP set was further reduced by applying population-wide polymorphism filters. The filtering included removal of SNPs with MAF lower than 5% and SNPs with more than 20% of missing genotypes across all breeds. MAF cutoff used for SNPs filtering was applied to the whole population (all breeds). This allowed to retain small proportion of SNPs that are monomorphic only in some breeds (presumably fixed for some reason, including selection and inbreeding). MAF value of 0.01 was used to characterize remaining SNP polymorphisms. SNPs deviating from HWE with critical *P*-value of 1.0E-06 in each breed separately were also removed resulting in a final panel of 43,923 common SNPs with average inter-marker distance of 55.7 kb (±78.0). The signals of diversifying selection were detected using pairwise Wright’s F_ST_ [18], the classical measure of population genetic differentiation. The F_ST_ values obtained for pairwise comparisons at each SNP were treated according to a methodology proposed by Akey et al. [19] and further applied by other studies [7]. In brief, standardized F_ST_ values were calculated (d_i_) as:

$$ {d}_i={\sum}_{j\ne i}\frac{F_{ST}^{ij}-E\left[{F}_{ST}^{ij}\right]}{sd\left[{F}_{ST}^{ij}\right]} $$

where $E\left[{F}_{ST}^{ij}\right]$ and $sd\left[{F}_{ST}^{ij}\right]$ denote the expected value and standard deviation of F_ST_ between breeds *i* and *j* calculated from all analyzed 43,923 SNPs. This allowed to make comparison of each breed against all other breeds under study. To account for stochasticity in locus-by-locus variation, a 10-SNP sliding window was further implemented on the obtained values. Candidate selected regions were then defined as the 99.9th percentile of the empirical distributions of window-averaged d_i_ values. The adjacent regions under selection were merged and (while searching for gene content) regions were expanded on both ends by 25 kb to detect neighboring, potentially linked genes.

The signals of positive selection within single breeds were detected using REHH statistics implemented in the Sweep v.1.1 software [6]. First, the obtained genotypes were phased and imputed using the fastPhase software [20]. The phased genotypes were then used to detect core haplotypes with minimum of three and no more than twenty SNPs. The detected longest non-overlapping core haplotypes were then subjected to EHH test, which is based on comparing a core haplotype with both higher frequency and higher EHH with other core haplotypes at the same *locus*. Subsequently, a probability that two randomly selected haplotypes within a core region are identical-by-descent for the entire interval spanning the core region to a given *locus* was computed [15, 21]. Finally, considering variation in recombination rates across the genome, the relative extended haplotype homozygosity (REHH) statistics was used [15] and calculated at about 1 cM (approximated to 1 Mb) distance [22] on both upstream and downstream directions (with exception of chromosome ends) from each core against all other cores within the region. To determine REHH significance, haplotypes were allocated to twenty frequency bins and the REHH values were compared between equally frequent core haplotypes found within the region. REHH *P*-values were ultimately obtained by a logarithmic transformation of the REHH values within these bins (to reach normality) and calculation of mean and standard deviation. The core haplotypes with the most extreme *P*-values (extended by 0.5 Mb in each direction) were filtered for frequency (> 0.25) and screened for overlapping pig ENSEMBL genes with the use of UCSC Genome Browser.

The functional annotation of detected genes was performed using the KOBAS 3.0 web server [23] and WebGestalt (WEB-based GEneSeTAnaLysis Toolkit) [24]. A gene list enrichment analysis was done according to all known pig genes applying a correction for multiple testing.

The population differentiation was additionally visualized using the principal component analysis (PCA) based on SNP genotypes and a cladogram of mean pairwise F_ST_ distances created using the neighbor joining (NJ) method [25].

## Results

### SNPs polymorphism parameters and breeds genetic differentiation

**Fig. 1:**
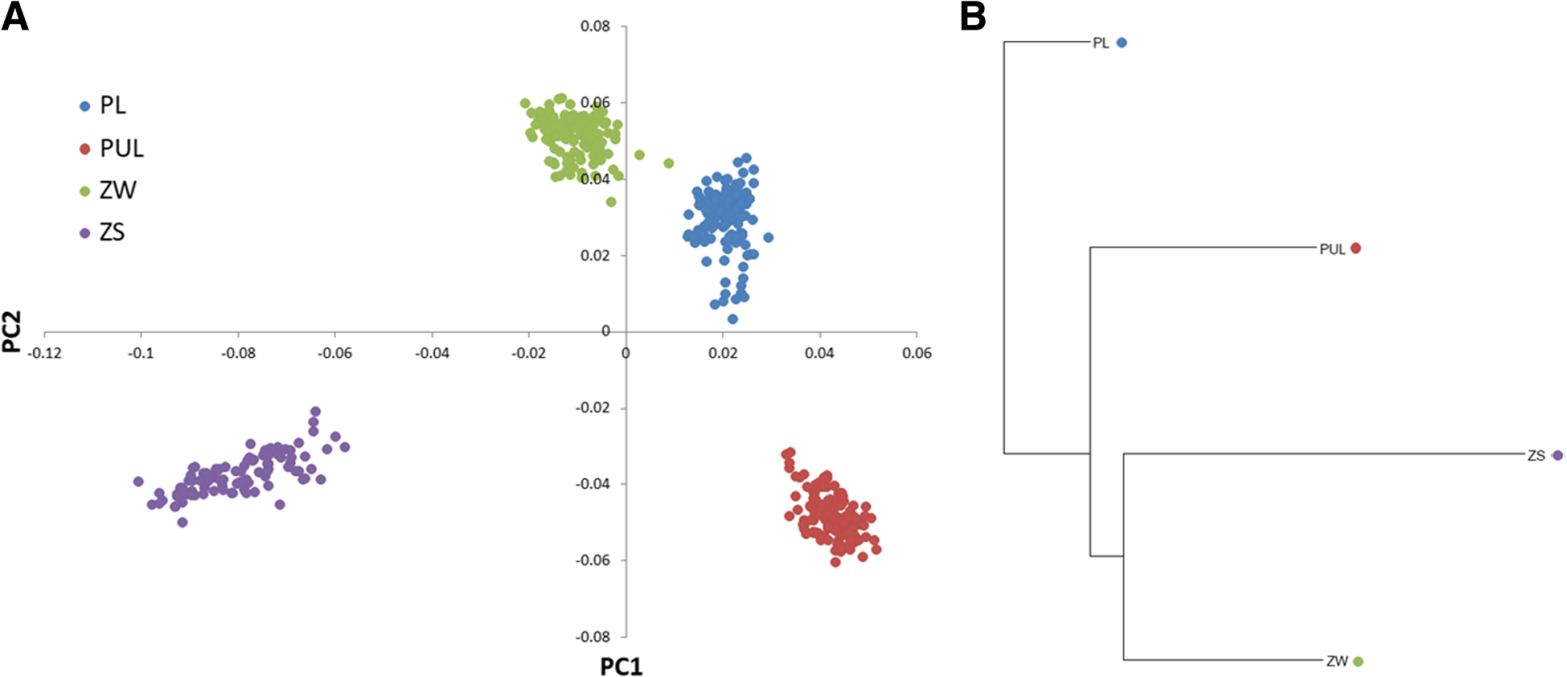
Genetic differentiation of the analyzed pig breeds based on (**a**) principal component analysis and (**b**) the neighbor joining method on mean pairwise F_ST_ distances

The applied SNPs filtering criteria allowed obtaining a common set of 43,923 SNPs polymorphic across the whole population with mean inter-marker distance of 55.7 kb (±78.0). The number of polymorphic SNPs (MAF > 0.01) per breed ranged from 37,423 to 43,567 in ZS and PL breeds, respectively. The average MAF across all SNPs was the lowest in ZS (0.212) and the highest in PL (0.279). The averaged observed heterozygosity per breed ranged from 0.3 to 0.367 for the same breeds (Table 1). Mean and weighted overall pairwise F_ST_ distances were the highest between PUL and ZS (0.143 and 0.173) and the lowest level of genetic differentiation was found between PL and ZW (0.085 and 0.097) (Table 2). The visualization of pig breeds genetic diversity using PCA and NJ methods is presented in Fig. 1.

**Table 1:** SNPs panel polymorphism parameters

Breed	SNPs No	Monomorphic SNPs (No)	Low frequency variants (0 < MAF < 0.01)	Polymorphic SNPs (MAF > 0.01)	Mean MAF	Observed heterozygosity
PL	43,923	157	199	43,567	0.279	0.367
PUL		288	71	43,564	0.274	0.361
ZW		1008	1254	41,661	0.251	0.344
ZS		4638	1862	37,423	0.212	0.300

**Table 2:** Mean (above diagonal) and weighted (below diagonal) pairwise F_ST_ distances between the studied pig breeds

Breed	PL	PUL	ZW	ZS
PL	–	0.0897	0.0853	0.1423
PUL	0.1014	–	0.1097	0.1432
ZW	0.0973	0.1276	–	0.1353
ZS	0.1731	0.1733	0.1685	–

### Signals of diversifying selection

**Fig. 2:**
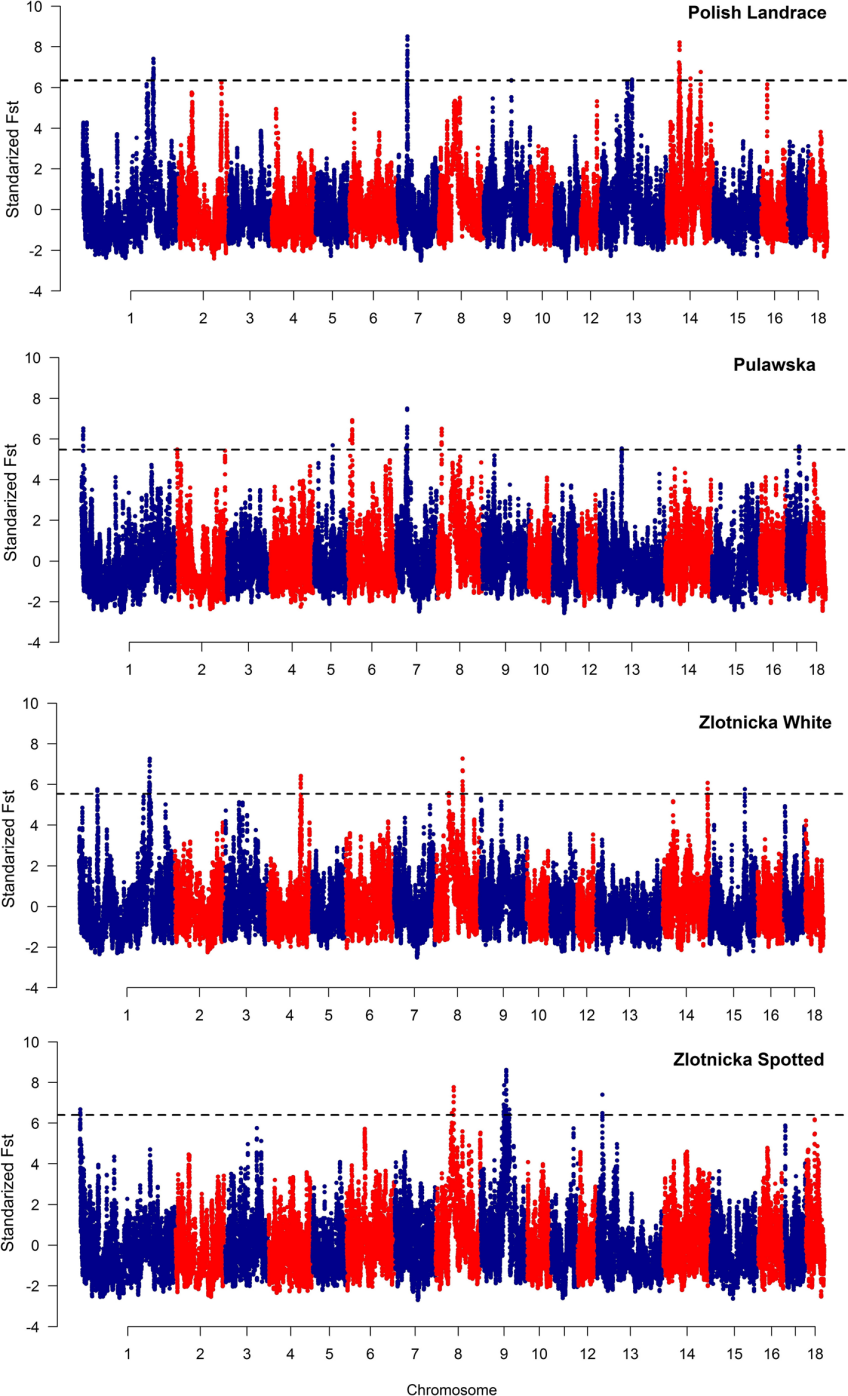
The genomic distribution of diversifying selection signals for all studied breeds. Dashed line indicates the top 0.1% of the highest standardized F_ST_ values

Signals of diversifying selection among the studied pig breeds were detected based on the breed-normalized pairwise F_ST_ distances (Additional file 2). After smoothing of the data by moving average, top 0.1% of the observations were considered as pinpointing breed-specific selection signals. After merging of overlapping signals, from 7 to 11 genome regions with strong selection signals were detected per breed with a size ranging from 266.8 kb to 2.9 Mb. The highest number of selection signals across all breeds was detected on SSC1, SSC7 and SSC14 (SSC9 only in ZS breed) and only single regions were detected on SSC2, 4, 5, 15 and 17. No signals (except closely positioned regions on SSC7, between 32 and 34 Mb of the genomic sequence in PL and PUL) were common for different breeds (Table 3, Fig. 2).

**Table 3:** Genome regions spanning the strongest detected diversifying (F_ST_-based) selection signatures

Breed	CHR	START	END	SIZE	START-25 kb	END+ 25 kb
PL	7	32,166,462	33,358,569	1,192,107	32,141,462	33,383,569
	13	104,557,045	105,298,541	741,496	104,532,045	105,323,541
	14	40,707,564	41,060,913	353,349	40,682,564	41,085,913
	14	41,333,642	41,977,281	643,639	41,308,642	42,002,281
	14	42,237,338	43,688,399	1,451,061	42,212,338	43,713,399
	14	78,434,877	78,742,346	307,469	78,409,877	78,767,346
	14	111,740,597	112,008,662	268,065	111,715,597	112,033,662
PUL	1	6,272,450	6,735,120	462,670	6,247,450	6,760,120
	2	365,309	1,264,220	898,911	340,309	1,289,220
	5	60,978,291	61,354,749	376,458	60,953,291	61,379,749
	6	7,872,494	8,303,476	430,982	7,847,494	8,328,476
	6	14,097,672	14,905,615	807,943	14,072,672	14,930,615
	7	33,416,020	33,720,562	304,542	33,391,020	33,745,562
	7	34,856,640	35,251,345	394,705	34,831,640	35,276,345
	7	35,935,629	37,260,234	1,324,605	35,910,629	37,285,234
	8	15,073,006	15,600,021	527,015	15,048,006	15,625,021
	13	75,723,927	76,244,239	520,312	75,698,927	76,269,239
	17	41,777,060	42,101,006	323,946	41,752,060	42,126,006
ZW	1	60,225,139	60,769,684	544,545	60,200,139	60,794,684
	1	229,502,611	229,792,874	290,263	229,477,611	229,817,874
	1	230,021,584	231,098,497	1,076,913	229,996,584	231,123,497
	1	231,365,442	232,552,969	1,187,527	231,340,442	232,577,969
	4	107,135,195	107,586,557	451,362	107,110,195	107,611,557
	8	45,191,096	47,560,329	2,369,233	45,166,096	47,585,329
	8	90,972,549	91,828,187	855,638	90,947,549	91,853,187
	14	146,952,943	147,444,441	491,498	146,927,943	147,469,441
	15	115,092,422	115,767,070	674,648	115,067,422	115,792,070
ZS	1	1,300,464	2,153,932	853,468	1,275,464	2,178,932
	8	52,565,396	55,529,571	2,964,175	52,540,396	55,554,571
	8	58,625,849	61,180,840	2,554,991	58,600,849	61,205,840
	9	75,593,700	75,973,705	380,005	75,568,700	75,998,705
	9	76,589,514	77,680,603	1,091,089	76,564,514	77,705,603
	9	78,073,285	78,534,222	460,937	78,048,285	78,559,222
	9	81,370,401	82,465,204	1,094,803	81,345,401	82,490,204
	9	82,868,790	84,766,063	1,897,273	82,843,790	84,791,063
	9	93,709,999	94,139,393	429,394	93,684,999	94,164,393
	13	17,239,351	17,506,216	266,865	17,214,351	17,531,216

To analyze gene content of the genome regions spanning the detected selection signals, each region was expanded by 25 kb on each end to account for potentially linked genes. This allowed for detection from 61 (ZS) to 116 (PUL) ENSEMBL genes per breed (Additional file 3). To manage variety of genes found within the selection signals we performed a functional analysis aimed at the identification of enriched processes. Keeping in mind that the detected selection signatures are associated with a number of phenotypic features differing the studied breeds (which are conditioned by complex and very different molecular mechanisms), we expected only very few genes connected with separate biological processes in the enrichment analysis and low statistical significance of the obtained results. This analysis was rather treated as a supporting method which helped us to manage the obtained extensive gene content. Nevertheless, this analysis allowed to reduce the complexity of the obtained data and allowed us to search for processes and underlying genes potentially being the targets of diversifying selection. The functional classification of well-annotated genes showed that the genes were mainly involved in GO biological processes connected with: metabolic processes, cellular processes, biological regulation, response to stimulus and developmental processes. Among the top ten enriched (pointwise *P* < 0.05) GO biological processes there were inter alia those connected with: lipid binding, fatty acid metabolic process, cellular senescence or response to muscle stretch. When functional classification was performed for individual breeds, visible differences in the enriched GO categories were detected (Table 4). In PL pigs, a large share of genes was engaged in metabolic pathways and lipid binding, and included e.g.: *COQ5, GATC, COX6A1, PLA2G1B, HK1, SDSL, PRIM2, ALDH2, SDS, GLTP* and *RPH3A* genes. In PUL breed, the genes involved in several GO categories were detected, including those connected with: striated muscle cell proliferation, skeletal muscle tissue regeneration (*PPARD* and *MAPK14*) and embryo implantation (*ARHGDIB, PPARD*). The diversifying selection signals in ZW breed encompassed genes enriching very general categories of biological processes, like e.g. transcriptional repressor activity, intracellular processes or processes in nuclear lumen, however, some of the genes were connected with more specific pathways like: tryptophan metabolism (*TDO2, AOX1*) or salivary secretion (*GUCY1A3, GUCY1B3*). In ZS breeds, selection signals were associated with genes responsible for e.g.: neuropeptide Y receptor activity and feeding behavior (*NPY1R, NPY5R*), metabolic processes (*PON1, PON2, PON3*) and cortisol metabolic process (*REST*).

**Table 4:** Top ten biological processes enriched in genes detected in the genome regions under diversifying selection in separate pig breeds

#Term	ID	Input number	Background number	*P*-Value	Corrected *P*-Value
PL					
Lipid binding	GO:0008289	5	150	0.0002	0.1501
Single-organism transport	GO:0044765	9	658	0.0004	0.1501
Cilium morphogenesis	GO:0060271	3	45	0.0006	0.1501
Single-organism localization	GO:1902578	9	708	0.0007	0.1501
Organic substance transport	GO:0071702	8	571	0.0007	0.1501
Macromolecule localization	GO:0033036	8	590	0.0009	0.1567
Regulation of DNA biosynthetic process	GO:2000278	2	14	0.0013	0.1574
Cell projection organization	GO:0030030	5	239	0.0014	0.1574
p53 binding	GO:0002039	2	15	0.0015	0.1574
Transport	GO:0006810	11	1158	0.0017	0.1574
PUL					
Skeletal muscle tissue regeneration	GO:0043403	2	9	0.0015	0.4425
Embryo implantation	GO:0007566	2	11	0.0021	0.4425
Tissue regeneration	GO:0042246	2	13	0.0027	0.4425
Cellular senescence	GO:0090398	2	14	0.0031	0.4425
Striated muscle cell proliferation	GO:0014855	2	16	0.0040	0.4425
Connective tissue development	GO:0061448	3	63	0.0051	0.4425
Regulation of myoblast differentiation	GO:0045661	2	19	0.0054	0.4425
Plasma membrane region	GO:0098590	4	133	0.0059	0.4425
Regeneration	GO:0031099	2	21	0.0064	0.4425
Cell aging	GO:0007569	2	22	0.0070	0.4425
ZW					
Transcriptional repressor activity, RNA polymerase II transcription factor binding	GO:0001191	2	24	0.0033	0.4411
Intracellular	GO:0005622	22	3854	0.0034	0.4411
Nuclear part	GO:0044428	9	989	0.0048	0.4411
RNA polymerase II transcription cofactor activity	GO:0001104	2	36	0.0069	0.4411
Nuclear lumen	GO:0031981	8	863	0.0071	0.4411
Transcription factor activity, RNA polymerase II transcription factor binding	GO:0001076	2	44	0.0100	0.4411
intracellular part	GO:0044424	20	3671	0.0101	0.4411
Nucleus	GO:0005634	12	1785	0.0121	0.4411
membrane-enclosed lumen	GO:0031974	8	971	0.0139	0.4411
intracellular organelle lumen	GO:0070013	8	971	0.0139	0.4411
ZS					
Neuropeptide Y receptor activity	GO:0004983	2	8	0.0001	0.0677
Neuropeptide receptor activity	GO:0008188	2	20	0.0007	0.1715
Feeding behavior	GO:0007631	2	31	0.0016	0.2289
Neuropeptide signaling pathway	GO:0007218	2	36	0.0021	0.2289
Peptide receptor activity	GO:0001653	2	64	0.0061	0.2289
G-protein coupled peptide receptor activity	GO:0008528	2	64	0.0061	0.2289
Circulatory system development	GO:0072359	3	218	0.0069	0.2289
Cardiovascular system development	GO:0072358	3	218	0.0069	0.2289
Cortisol metabolic process	GO:0034650	1	5	0.0106	0.2289
Response to electrical stimulus	GO:0051602	1	5	0.0106	0.2289

### Selection signals between breeds with different phenotypical features

To detect genome regions bearing variants potentially responsible for the most pronounced phenotypical differences between breeds, the pig breeds with white (PL, ZW) and spotted (PUL, ZS) coat color patterns were compared using F_ST_ statistics. The comparison between single color and spotted pig breeds revealed the strongest (99.9th percentile of the observations) selection signals on SSC3, SSSC7, SSC8 and SSC9 (Table 5). The most pronounced selection signal, involving five separate regions (between 50.5 and 58.6 Mb of sequence) was located on SSC8 in close vicinity to the *KIT* gene *locus* (SSC8, 41.4–41.5 Mb). Altogether, the regions spanned 72 different ENSEMBL genes which together did not enrich any biological processes of pathways, however, again included *PPARD* gene.

**Table 5:** Genomic regions spanning the strongest detected diversifying selection signals between single-color and spotted pig breeds

CHR	START	END	SIZE	START-25 kb	END+ 25 kb
3	110,615,540	111,359,515	743,975	110,590,540	111,384,515
7	32,456,532	33,142,614	686,082	32,431,532	33,167,614
8	50,537,893	52,513,888	1,975,995	50,512,893	52,538,888
8	51,235,665	53,929,233	2,693,568	51,210,665	53,954,233
8	52,301,582	55,181,102	2,879,520	52,276,582	55,206,102
8	52,565,396	55,647,917	3,082,521	52,540,396	55,672,917
8	58,598,344	61,863,927	3,265,583	58,573,344	61,888,927
8	69,065,977	71,405,594	2,339,617	69,040,977	71,430,594
9	90,863,475	91,514,134	650,659	90,838,475	91,539,134

### Within-breed selection signatures

**Fig. 3:**
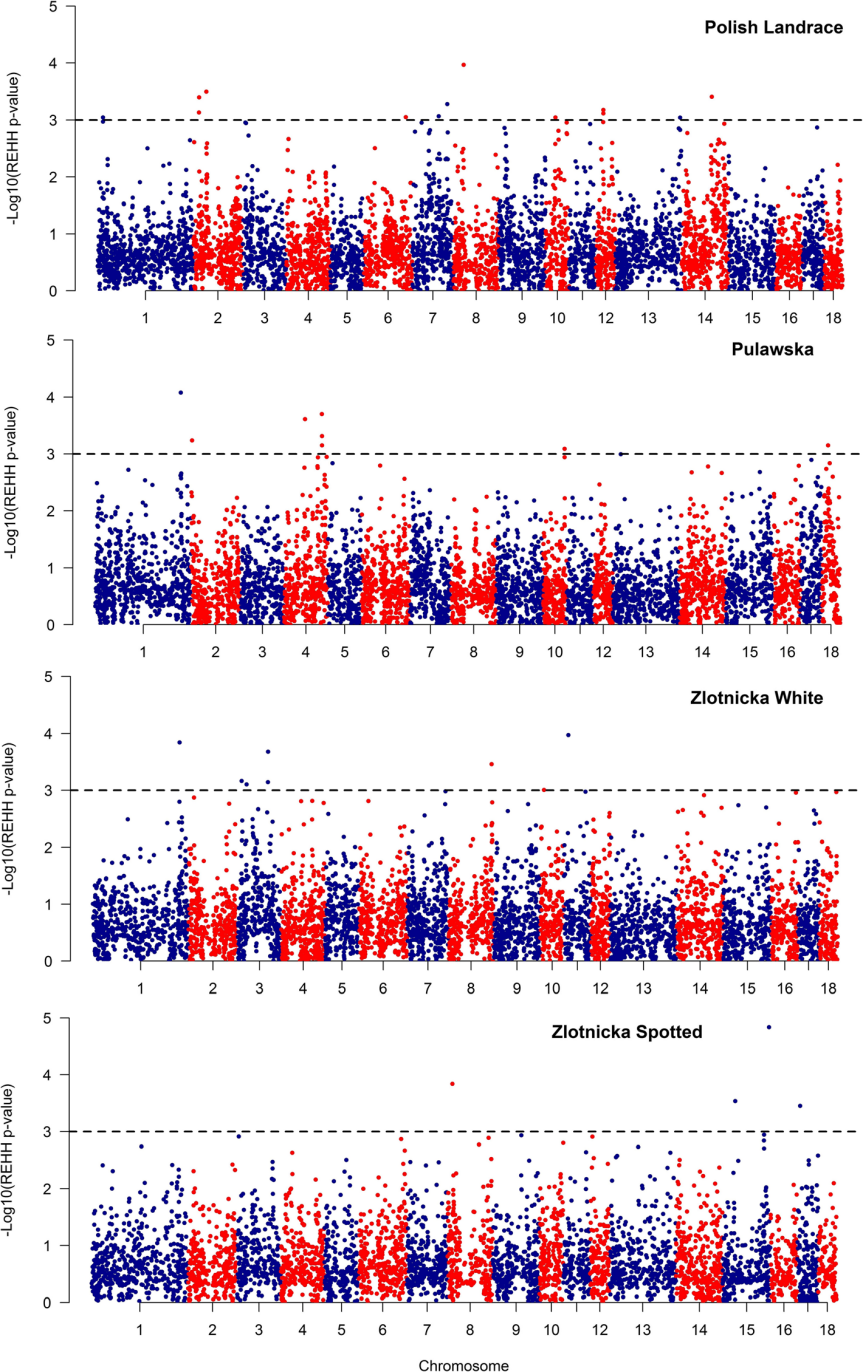
The genomic map of REHH *P*-values for all core haplotypes showing a frequency > 0.25. Dashed line represents the significance threshold of 0.001

The analysis performed with the Sweep v.1.1 software allowed detection from 4546 (ZS) to 5638 (PUL) longest non-overlapping core regions (CR – a unique genome region spanning *locus* specific core haplotypes) consisting of minimum three and no more than 20 SNPs with average lengths ranging from 193.7 kb (PL) to 394.3 kb (ZS). For all these core haplotypes, from 37,809 (ZS) to 46,162 (PUL) EHH tests were performed – from 8.2 to 8.3 per core region, on average (Additional file 4). Considering that alleles being under positive selection are fixed or going to be fixed and hence core haplotypes harboring these alleles should be frequent [21], core haplotypes with the frequency < 0.25 were removed. This allowed selection from 14,079 (ZS) to 17,580 (PUL) REHH tests per breed for which from 118 (ZS) to 176 (PL) core haplotypes were outliers at the significance level of 0.01. The obtained REHH *P*-values were –log10 transformed and plotted against the chromosomal positions to visualize outlying core haplotypes and selection patterns across the breeds genome (Fig. 3, Additional file 5). This analysis showed clear non-random distribution of selection signals across the breeds genome with visible overrepresentation of long and common haplotypes on e.g.: SSC2 and 14 in PL or SSC4 and 18 in PUL breed. The selection patterns also clearly differed between the analyzed breeds. To minimize the number of genes potentially associated with significant core haplotypes, only cores with P-value lower than 0.001 (representing the strongest selection signals) were further analyzed. The positions, haplotype frequency and other statistics for the most significant haplotypes belonging to separate core regions are presented in Table 6.

**Table 6:** Statistics for the most significant core haplotypes being under selection and belonging to separate core regions in the analyzed breeds

Breed	Chr	Start Base	End Base	Size (kb)	Hap frequency	EHH	REHH	REHH *P*-Value	Genes directly overlapping with core haplotypes^a^
PL	1	17,474,143	17,532,523	58.38	0.33	0.77	10.45	0.000902	*LOC100152956, LOC100621885, LOC100512136, HOXB2, ZNF22, LOC100739279, C10orf10, RASSF4, LOC100157415, HOXB1, ALG6, HOXB3, ALOX5, LOC100523276, HOXB4, MARCH8*
	2	18,722,196	18,775,121	52.925	0.31	0.53	10.91	0.00074	
	2	18,850,395	18,872,659	22.264	0.31	0.45	12.46	0.0004	
	2	42,642,528	42,708,543	66.015	0.64	0.60	9.68	0.000318	
	6	137,335,517	137,534,028	198.511	0.27	0.79	10.35	0.000892	
	7	87,029,852	87,066,927	37.075	0.59	0.88	7.79	0.000858	
	7	115,164,638	115,233,642	69.004	0.39	0.89	10.21	0.000526	
	8	33,740,426	33,760,858	20.432	0.34	0.88	16.21	0.000108	
	10	32,485,201	32,589,926	104.725	0.60	0.53	7.74	0.000902	
	12	24,427,829	24,643,031	215.202	0.27	0.64	11.03	0.000668	
	12	24,696,740	24,812,751	116.011	0.27	0.64	10.70	0.000768	
	13	211,907,795	211,989,807	82.012	0.48	0.48	9.93	0.00091	
	14	98,887,138	99,642,277	755.139	0.42	0.93	10.94	0.00039	
PUL	1	279,545,606	279,701,487	155.881	0.52	0.83	5.87	0.000374	*PRKCQ, TERF1, FAM71F2, SBSPON, IMPDH1, LRP5*
	1	279,545,606	279,701,487	155.881	0.52	0.74	7.52	8.35E-05	
	2	3,166,230	3,236,832	70.602	0.58	0.58	5.09	0.000578	
	4	68,321,268	68,385,012	63.744	0.58	0.63	5.93	0.000245	
	4	123,472,417	123,553,728	81.311	0.48	0.99	7.18	0.000199	
	4	123,712,634	123,819,209	106.575	0.48	0.92	5.73	0.000706	
	4	123,838,086	123,907,205	69.119	0.48	0.90	6.14	0.000485	
	10	70,638,252	70,669,410	31.158	0.35	0.52	8.00	0.000812	
	18	20,991,435	21,077,884	86.449	0.77	0.55	4.22	0.00071	
ZW	1	283,594,245	283,667,732	73.487	0.93	0.65	2.91	0.000144	*C1orf115, SUSD1, PRKCE, MARC2*
	3	14,090,668	14,221,604	130.936	0.32	0.98	6.04	0.000686	
	3	30,457,204	30,733,237	276.033	0.45	1.00	5.01	0.000792	
	3	100,417,119	100,448,894	31.775	0.81	0.55	2.95	0.000719	
	3	100,521,767	100,536,654	14.887	0.71	0.69	4.08	0.000211	
	3	100,521,767	100,536,654	14.887	0.71	0.70	3.88	0.000295	
	8	141,594,565	141,644,208	49.643	0.46	0.80	5.73	0.000349	
	8	141,594,565	141,644,208	49.643	0.46	0.98	5.49	0.000457	
	10	12,274,858	12,388,946	114.088	0.27	1.00	5.98	0.000988	
	11	14,256,201	14,501,882	245.681	0.32	1.00	8.03	0.000107	
ZS	8	16,617,477	16,681,729	64.252	0.32	1.00	6.07	0.000145	*UBE2F, RAMP1*
	15	41,476,357	41,710,320	233.963	0.36	1.00	4.98	0.000291	
	15	151,930,390	152,024,615	94.225	0.39	0.95	7.12	1.46E-05	
	17	9,863,762	9,926,558	62.796	0.27	0.64	5.67	0.000355	

^a^genes with unassigned name were removed from the table

After extension of the detected significant (*P* < 0.001) core haplotypes to 1 cM on both ends, 171 unique genes potentially being under selection were found in PL, 116 in PUL, 84 in ZW and 35 in ZS (Additional file 6). The functional annotation of the genes allowed credible GO categories enrichment analysis only for PL and PUL pigs, with only single genes connected with separate GO categories in ZW and ZS pigs (Table 7). Nevertheless, among the top 10 enriched biological processes, the genes associated inter alia with skeletal system development and morphogenesis (*HOXB1, − 2, − 5, − 6, − 7, − 9*) were found in PL pigs and regulation of protein transport and localization (*AIP, TBC1D10C, GCC1, LEP*) or muscle fiber development (*FLNC, SMO*) in PUL pigs. In ZW breed the suggestive biological processes were involved in e.g.: metal ion homeostasis and regulation (*TTC7A, SLC30A10*), negative regulation of innate immune response (*DUSP10*) and negative regulation of T cell apoptotic process (*SLC46A2*). In ZS breed, the most commonly represented processes included: galactosidase/galactosidase activity and lipid catabolic process (*LOC102167689, LOC102167689*).

**Table 7:** Top ten biological processes enriched in genes detected in the core haplotypes under positive selection in individual pig breeds

#Term	ID	Input number	Background number	*P*-Value	Corrected *P*-Value
PL					
Embryonic skeletal system development	GO:0048706	6	37	8.68E-07	0.001
Embryonic skeletal system morphogenesis	GO:0048704	5	29	5.69E-06	0.0037
Skeletal system morphogenesis	GO:0048705	6	65	1.71E-05	0.0074
Anterior/posterior pattern specification	GO:0009952	6	70	2.53E-05	0.0081
Regionalization	GO:0003002	7	118	4.95E-05	0.0127
Skeletal system development	GO:0001501	7	123	6.35E-05	0.0136
Pattern specification process	GO:0007389	7	131	9.26E-05	0.0170
Sequence-specific DNA binding	GO:0043565	10	305	0.00015	0.0251
Chordate embryonic development	GO:0043009	7	171	0.0004	0.0603
Embryo development ending in birth or egg hatching	GO:0009792	7	173	0.0005	0.0603
PUL					
Regulation of hair cycle	GO:0042634	2	5	0.0005	0.2658
Establishment of protein localization to organelle	GO:0072594	5	151	0.0010	0.2658
Regulation of protein import	GO:1904589	3	41	0.0013	0.2658
Regulation of protein import into nucleus	GO:0042306	3	41	0.0013	0.2658
Protein targeting	GO:0006605	5	162	0.0014	0.2658
Regulation of nucleocytoplasmic transport	GO:0046822	3	46	0.0018	0.2658
Regulation of protein localization to nucleus	GO:1900180	3	49	0.0021	0.2658
Negative regulation of peptide hormone secretion	GO:0090278	2	13	0.0024	0.2658
Negative regulation of peptide secretion	GO:0002792	2	13	0.0024	0.2658
Muscle fiber development	GO:0048747	2	14	0.0028	0.2658
ZW					
Cellular transition metal ion homeostasis	GO:0046916	2	26	0.0032	0.4776
Transition metal ion homeostasis	GO:0055076	2	35	0.0056	0.4776
Nucleotidase activity	GO:0008252	1	5	0.0180	0.4776
Zinc ion homeostasis	GO:0055069	1	6	0.0210	0.4776
Negative regulation of innate immune response	GO:0045824	1	6	0.0210	0.4776
Glycosphingolipid biosynthetic process	GO:0006688	1	6	0.0210	0.4776
Glucosyltransferase activity	GO:0046527	1	6	0.0210	0.4776
Negative regulation of T cell apoptotic process	GO:0070233	1	6	0.0210	0.4776
Cellular zinc ion homeostasis	GO:0006882	1	6	0.0210	0.4776
Response to zinc ion	GO:0010043	1	6	0.0210	0.4776
ZS					
Galactosidase activity	GO:0015925	1	5	0.0087	0.3709
Sphingolipid catabolic process	GO:0030149	1	5	0.0087	0.3709
Membrane lipid catabolic process	GO:0046466	1	5	0.0087	0.3709
Glucosidase activity	GO:0015926	1	5	0.0087	0.3709
Activation of NF-kappaB-inducing kinase activity	GO:0007250	1	6	0.0101	0.3709
snRNA processing	GO:0016180	1	7	0.012	0.3709
Postreplication repair	GO:0006301	1	8	0.0130	0.3709
snRNA metabolic process	GO:0016073	1	9	0.0144	0.3709
Positive regulation of NIK/NF-kappaB signaling	GO:1901224	1	9	0.0144	0.3709
Protein K63-linked ubiquitination	GO:0070534	1	10	0.0159	0.3709

## Discussion

Natural or artificial selection are the major mechanisms driving differentiation of the populations. Pig domestication resulted in considerable changes in the phenotypes and behavior of the animals. In the early stages of domestication, unconscious selection for behavioral traits was applied and this early stage was followed by methodical selection in which specific traits were selected based on breeding goals [26, 27]. This resulted in development of specialized breeds, improved to produce desired animal products or to represent a desired morphological standard. The artificial selection increased the variation between domesticated animals and their wild ancestors and generated a variety of different populations, differing in phenotypical features related to their specialization [28]. Identifying recent positive selection signatures in domesticated animals can provide valuable information on genomic regions that are under the influence of both artificial and natural selection, and thus, can help to identify beneficial variants and underlying biological pathways influencing economically important traits.

In this study, by using two largely complementary methods we detected selection signatures in four pig breeds, including Landrace and three conserved native breeds representing a maternal breeding component. The native pig populations can be considered as unselected, because the conservation programs are more focused on preserving genetic diversity and breed standard than improvement of production traits. The analysis of breeds’ genetic differentiation based on a cladogram obtained with the neighbor-joining method on the averaged F_ST_ values (accounting for all pairwise distances) showed the highest genetic similarity between both Złotnicka pig breeds. Such result was expected, due to the fact that both breeds originated from the same geographic background and were created in a similar time period based on the same genetic group. The clear separation of Landrace from the Polish conserved breeds was also observed, which probably reflects its origin from German and Swedish pig breeds and its high speciation in terms of production. The applied PCA method showed the highest genetic similarity between Polish Landrace and Złotnicka White pigs which both represent white, meat-type pigs with dropping ears.

In the studied pig breeds, we detected several selection signals, representing genome regions that were differentially fixed in different breeds or representing within-breed selection signatures. The diversifying selection signals were detected based on the comparison of a specific breed with all other breeds under study, presuming that these signals will be characteristic for unique breed features or strongly fixed breed traits being poorly developed in the other compared breeds. This can be an explanation for the lack of selection signals being common for different breeds. With the F_ST_-based method, we detected large (over 1 Mb in size) selection sweeps on SSC7 (32.1–33.3 Mb) and 14 (42.2–43.7 Mb) among the top 0.1% of strongest selection signals in Polish Landrace. Several selection signals associated with these chromosomes were previously reported in other pig breeds [7, 29], however, their direct comparison is difficult since diversifying section signals are strongly dependent on structure of breeds used for comparisons. Several GWAS showed the association of closely positioned SNPs on SSC7 with backfat thickness [30], fatty acid metabolic indices [31], number of teats or body weight and height [32]. A similar to ours region on SSC14 was shown to be associated with a number of teats [33, 34]. The animals of conserved breeds included in this study considerably differed in terms of this trait, ranging from on average 13.71 (±0.97) teats in ZS to 14.22 (±0.88) in PUL breed. Unfortunately, no reproductive performance records were available for the PL pigs analyzed in this study. Nevertheless, according to the national evaluation, the average number of teats in PL breed is visibly higher than this observed in the conserved breeds - 15.02 (Additional file 7). This suggests that the detected strong selection signals on these autosomes may be a result of long-term selection for conformation and reproductive performance traits in PL breed.

While screening detected selection signals for gene content we extended the detected regions by additional bases. Extension of the detected regions was motivated by a need to account for linkage disequilibrium and the applied sliding-window approach. While moving average smooths the data, some surrounding genes may not fall directly into the selection signals. Because of some accidental differences in allele frequencies (e.g. genetic drift), selection signal peaks can be slightly shifted in relation to real functional variant locations. To not to lose this information we extended F_ST_ - based regions by additional 25 kb, which is an approximated half of mean distance between SNPs in this study. In the REHH method we extended the detected signals by 0.5 Mb in each direction. In our data, we found that core haplotype domains (their extended homozygosity) on average spans over 0.7 Mb in upstream and downstream directions from cores. We have narrowed these regions by adding 0.5 Mb to each core haplotype to account only for genes within the region of relatively high linkage with the core SNPs. Similar approach was previously proposed by Qanbari et al. [21]. The size of selection signals detected using the two approaches was comparable, suggesting that thanks to the linkage disequilibrium, selection signals are extensive and should not be narrowed down only to the detected signal peaks.

Within the strongest differential (F_ST_-based) selection signals detected in Polish Landrace we found several candidate genes which may be responsible for the well-developed breed characteristics like good fertility and high growth rate. One of the interesting detected genes is *RASAL1* gene (RAS Protein Activator Like 1), acting as a suppressor of RAS function. The protein enhances the weak intrinsic GTPase activity of RAS proteins resulting in the inactive GDP-bound form of RAS, thereby allowing control of cellular proliferation and differentiation [35]. A large share of detected genes in PL breed was engaged in metabolic pathways and included e.g. *HK1* (Hexokinase 1) gene which phosphorylates glucose to produce glucose-6-phosphate which is the first step in glucose metabolism pathways. Glucose metabolism is important for energy homeostasis but has also an effect on growth and meat quality traits [36]. Two other genes were also detected within the identified selection signals in PL pigs, such as: *LC8* (LC8 dynein light chain – *DYNLL1*) and *KHDRBS2* (KH RNA Binding Domain Containing, Signal Transduction Associated 2) – related with reproduction traits. In mice, it was established that dynein regulates meiotic checkpoint during oocyte maturation [37] and *KHDRBS2* was found to be associated with the number of teats in the GWA study in Large White pigs [33]. The other gene – *PRIM2* (which encodes a DNA primase – large subunit) – was previously found in Bayesian GWAS to be in a gene network associated both with the number of stillborn piglets (SB) and the number of teats (NT) [33].

Among the strongest diversifying selection signals in PUL pig breed, we detected one with length over 1 Mb on SSC7 (35.9–37.3 Mb). This region was previously described to bear several QTLs for health, meat and carcass traits and exterior (Pig QTLdb, [38]). Within all diversifying selection signals in PUL pigs we detected candidate genes associated with inter alia striated muscle cell proliferation, skeletal muscle tissue regeneration (*PPARD* and *MAPK14 -* GO:0043403) and embryo implantation (*ARHGDIB, PPARD* - GO:0007566) important for fertility traits. The most interesting diversifying selection candidate in this breed seems to be *PPARD* gene (Peroxisome proliferator-activated receptor beta/delta) located on SSC7, which was shown to be associated both with ear morphology and backfat thickness [39, 40]. A previous study identified a missense mutation in the *PPARD* gene that significantly reduces its transcription activity, and consequently causes enlarged external ears in pigs [40, 41]. This is concordant with the phenotype of PUL breed which is the only breed with prick ear morphology in this study and the detected signal potentially results from diversifying selection for ear morphology among the studied breeds.

In the ZW breed, large size sweeps were detected on SSC1 (230.0–232.5 Mb) and 8 (45.2–47.5 Mb). These regions were previously shown to carry mainly QTLs for meat and carcass traits (SSC1) or health (SSC8) (Pig QTLdb, [38]). Within the strongest diversifying section signals in this breed, we detected genes associated inter alia with tryptophan metabolism (*TDO2, AOX1*) or salivary secretion (*GUCY1A3, GUCY1B3*). Tryptophan (TRP), as a precursor of neurotransmitters, was shown to be involved in pale, soft, exudative (PSE) pork syndrome [42] and dietary TRP deficiency was shown to correlate with decline of the appetite leading to reduced growth performance [43]. The TRP metabolism-associated genes may be then related with high stress resistance reported in the ZW breed and suggest its potential association with good meat quality and growth in this breed.

The largest genomic regions associated with differential selection in ZS breed were detected on SSC9. The regions were located between 75.6 Mb and 84.8 Mb of the chromosome sequence and were previously shown to carry mainly QTLs for exterior and reproduction features (Pig QTLdb, [38]). In ZS breed, the strongest detected selection signals were associated e.g. with genes responsible for: neuropeptide Y receptor activity and feeding behavior (*NPY1R, NPY5R*), metabolic processes (*PON1, PON2, PON3*) and cortisol metabolic process (*REST*). Our interest focused especially on genes associated with neuropeptide Y (NPY) activity. NPY has been implicated in several human diseases involving fat deposition aberrations and obesity [44]. This has special importance taking into account that Złotnicka Spotted breed is characterized by relatively high fat content in the carcass and, in comparison to other breeds, the meat of the ZS pigs is characterized by specific marbling resulting from higher intra muscular fat content. It was found that NPY reduces energy expenditure by decreasing adipose tissue thermogenesis [45, 46] and the obesity of mice was attenuated when NPY was knocked out [47, 48]. NPY deficiency also impaired responses to a palatable high fat diet in mice [49] and animals in which proopiomelanocortin neurons were knocked out were obese and hyperphagic [50–52]. These studies show the strong association of NPY with body fat generation by acting through several NYP receptors and suggest that two genes detected in this study that code for NPY receptors (2 and 5) may be connected with higher fat content and general higher fatness in the ZS breed when compared to the other studied breeds. The analysis of average back-fat thickens in the studied ZS animals confirmed general observations on this breed and showed that the average back-fat thickens was statistically significantly higher (*P* < 0.001) in ZS pigs (23.13 ± 3.53) than in three other analyzed breeds (in a range of 9.27 in PL to 18.03 in ZW breed) (Additional file 7).

Our F_ST_-based comparison of white and spotted pig breeds revealed an ambiguous selection signal in SSC8 positioned in close vicinity to *KIT* gene – a known coat color modulator [53]. However, a few other strong signals were detected on SSC3, SSSC7 and SSC9, which did not overlap with any of genes commonly implicated in coat pattern in mammals, like e.g.: *EDNRB, KITLG* or *MC1R* [53]. Nevertheless, in a previous work similar to ours, QTL regions on chromosomes 7 and 9 were detected [54] which were shown to have effects on coat color extension in crossbreed pigs. Significant QTL *loci* affecting black spotting were also detected on SSC3 and SSC9 in exotic pig crosses [55]. This suggests that at least a few other coat color-related variants can be present in the studied pigs’ genomes. Another explanation of the observed signatures pattern can be that the phenotypes of both studied spotted breeds (PUL and ZS) are not yet fully stabilized or the combination of phenotypes used for comparisons was not fully correct, because PUL pigs can have black or reddish spots on slightly pigmented skin, whereas no skin pigmentation is characteristic for the other studied breeds, which may bias the obtained results.

**Fig. 4:**
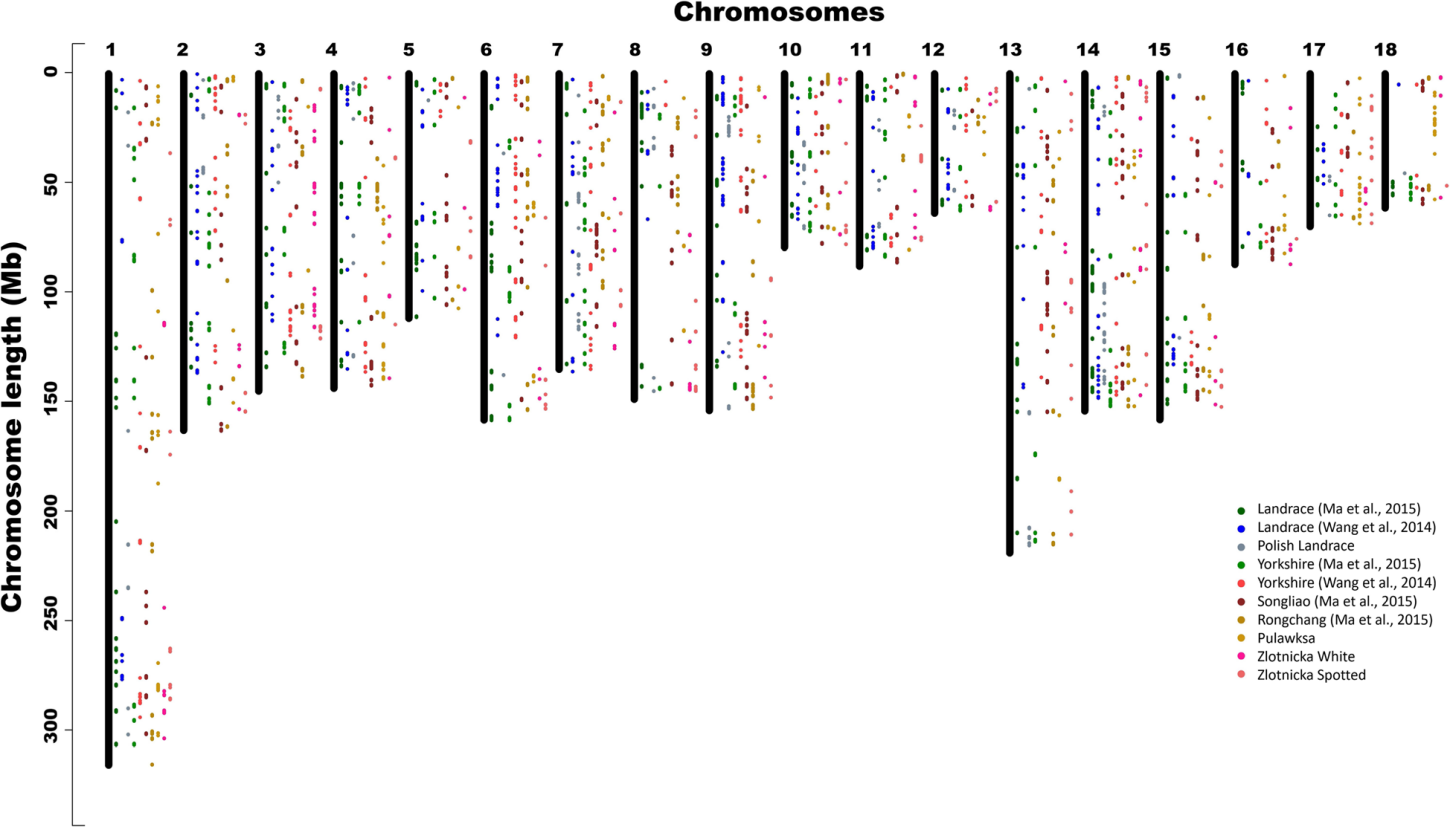
The comparison of the detected, significant (*P* < 0.01) within-breed selection signatures with previous results obtained using similar methods in different breeds

At least some of the detected divergent selection signals could be easily associated with genes potentially responsible for traits especially pronounced or fixed in the studied breeds and suggest the detection of genome regions connected with mechanisms underlying differentiating traits. To supplement this data with information on within-breed selection signatures, which are independent of breeds differentiation, we additionally used REHH statistics [15], which was shown to be the most powerful for detecting ongoing selection for which the target allele has a moderate to high frequency [56]. The use of such data allowed us to search for selection signals connected with traits that are selected across all studied breeds, representing variants that are still segregating and are under ongoing selection. With this approach we detected from 118 (ZS) to 176 (PL) core haplotypes per breed being the outliers at the significance level of 0.01. The general genomic pattern of these signals had visible similarity among the studied breeds and was comparable with previously analyzed pig breeds (Fig. 4). The visible clusters of strong selection signals were found on several chromosomes, however, the particularly visible were those located in distal parts of SSC1, SSC14 and SSC15 (Fig. 4). These chromosomal regions were previously associated with meat color density (SSC14) [3], growth, reproduction and immune responses (SSC1, SSC15) [29], which seems reasonable as most of pig breeds are selected towards the improvement of these traits.

To analyze in details the genomic regions with the strongest signs of selection, we took a closer look at regions bearing haplotypes with *P*-value lower than 0.001. After extending them to 1 cM on both sides, we detected from 35 (ZS) to 171 (PL) unique genes per breed potentially being under positive selection. Several of those genes are connected with important production features like growth and fertility. Exemplarily, in PL pigs we identified 10 genes belonging to the Hox family (*HOX1* to *HOX9* and *HOX13*), which are key factors in the implantation and development of the embryo and regulate the function of the endometrium [57–62]. In PL pigs, we also detected a gene belonging to the ESR receptor signaling pathway – *STAT5* – responsible for the signal transduction from the estrogen receptor. The *STAT5* gene itself is broadly tested for association with fertility and milk production traits [63–65]. The STAT5 protein plays a key role in signaling in the estrogen receptor pathway and therefore can play an important role in modulating the role of estrogen [66]. A gene encoding the estrogen receptor itself – ER – recognized as a key component responsible for female reproduction traits [67] and reproductive-related behavior in female [68] was also detected within the selection signatures found in the studied PL pigs. Furthermore, our analysis allowed identification of *CXCL12* gene (C-X-C Motif Chemokine Ligand 12) which as a ligand for the G-protein coupled receptor plays an important role in many biological processes including embryogenesis [69]. In pigs, *CXCL12* gene was identified as one of differentially expressed genes up-regulated in endometrium at 18th day of pregnancy, which can indicate its potential role in embryogenesis [70].

In the regions overlapping with the strongest within-breed selection signals in PUL breed as many as seven genes involved in lipid metabolism were detected (*DHCR7, CLPS, PNPLA1, PPARD, BPIFB5, CLPSL2, BPIFB1*) which may be associated with former selection of this breed toward reduced fat content in carcass. We also detected genes connected with immune system processes related to resistance to bacterial (*FLNC, TCIRG1*) and virus (*POLD4, SND1*) diseases, which fits to the characteristics of this native breed which is known to have a good disease resistance and adaptation to harsh environmental conditions. In Złotnicka White pigs, the strongest within-breed selection signals encompassed genes associated with inter alia the immune system processes and adaptation to environmental conditions or stress response. The first group of genes included those connected with inflammation mediated by chemokine and cytokine: *GNG10, PRKCE, SOCS5* or inflammatory mediator regulation of TRP channels – *MAPK10* (mitogen-activated protein kinase 10) and *PRKCE* (protein kinase C epsilon), which may contribute to the good health and disease resistance observed in this breed. The second group included *MCFD2* gene engaged in response to a stimulus and other genes responsible for response to stress (GO:00069 50)/stress-activated MAPK cascade, like: *MARC1, ERCC4, DUSP10, MAPK10*.

In Złotnicka Spotted breed, some predicted genes involved in lipid and carbohydrates metabolism were detected and were engaged in processes connected with: sphingolipid catabolism (*LOC102167689*), membrane lipid catabolism or glucosidase/galactosidase activity (*LOC102167689*) which as metabolic processes are often associated with selection signals in pigs [3]. The detection of the *SCLY* gene encoding isoform X1 of selenocysteine lyase associated with selenium metabolism and viral mRNA translation seems to be interesting because of the fact that it was established that SCLY selenocysteine plays a key role in lipid metabolism and can be related to selenium concentration in meat [71].

## Conclusions

Summarizing, in this study we used two different but largely complementary statistical approaches, REHH and pairwise F_ST_, to detect selection signatures in four Polish pig breeds, including three native unselected breeds. F_ST_-derived signatures were useful to detect diversifying selection signals across breeds (represented by *loci* for which alleles were differentially fixed in different breeds) and allowed us to indicate genes connected e.g. with fertility, growth and metabolism which are potentially responsible for phenotypes of the studied breeds. These selection signals also comprised *PPARD* gene that contributes to the shape of external ears in pigs and genes encoding neuropeptide Y receptors involved in fat deposition and stress response which are important features differentiating the studied breeds. REHH statistics pointed several within-breed selection signals overlapping with genes connected with a broad range of functions, including e.g.: metabolic pathways, immune system response or implantation and development of the embryo. The study provides many potential candidate genes responsible for traits selected in the individual breeds and gives a strong basis for further studies aimed at closing the gap between the genotype and phenotype of the studied pig breeds.

## Additional files

Additional file 1: Breed characteristics. (DOCX 12 kb)

Additional file 2: Standardized F_ST_ values (di) for all studied breeds averaged within ten-SNP sliding windows. (XLSX 4722 kb)

Additional file 3: Genes detected within the strongest diversifying selection signals detected in the studied pig breeds. (XLSX 71 kb)

Additional file 4: Summary characteristics of core regions (CR) or core haplotypes (CH) in the studied pig breeds. (XLSX 13 kb)

Additional file 5: REHH *P*-values for the detected core haplotypes with the highest frequency. (XLSX 730 kb)

Additional file 6: Genes detected within the strongest within-breed selection signals detected in the studied pig breeds. (XLSX 99 kb)

Additional file 7: Comparison of selected phenotypic traits for the studied pig breeds. (DOCX 19 kb)

## Abbreviations

cM: Centimorgan
GWAS: Genome-wide association study
HWE: Hardy-Weinberg equilibrium
LD: Linkage disequilibrium
MAF: Minor allele frequency
PCA: Principal component analysis
PL: Polish Landrace
PUL: Puławska pig
QTL: Quantitative trait locus
REHH: Relative extended haplotype homozygosity
SNP: Single nucleotide polymorphism
SSC: *Sus scrofa* (chromosome)
ZS: Złotnicka Spotted pig
ZW: Złotnicka White pig
