# Supplementary material for: A genome-wide detection of selection signatures in conserved and commercial pig breeds maintained in Poland
Source: BMC Genet. 2018 Oct 22;19:95. doi: 10.1186/s12863-018-0681-0 (PMC6198424; doi:10.1186/s12863-018-0681-0)
Supplement: Supplementary file 1 — Breed characteristics. (DOCX 12 kb) [file 12863_2018_681_MOESM1_ESM.docx]

Additional File 1. Breed characteristics

| Breed | Coat color | Ear morphology | Type | Component | Growth rate  g/day (Sex) | Puberty |
| --- | --- | --- | --- | --- | --- | --- |
| PL | White | Dropping (floppy or narrow and half-stiff | meat | Maternal | Fast  643g (F) 714g (M) | Early |
| PUL | Spotted (black or reddish spots on white/grey pigmented skin) | Prick (slightly inclined forwards) | meat-fat | Maternal | Medium-fast  565g (F) 576g (M) | Early |
| ZW | white | Dropping | meat-fat | Maternal | Slow  488g (F) 462g (M) | Late |
| ZS | Spotted (black spots on white skin) | Dropping | meat-lard | Maternal | Slow  440g (F) 437g (M) | Late |
