# Supplementary material for: A genome-wide detection of selection signatures in conserved and commercial pig breeds maintained in Poland
Source: BMC Genet. 2018 Oct 22;19:95. doi: 10.1186/s12863-018-0681-0 (PMC6198424; doi:10.1186/s12863-018-0681-0)
Supplement: Supplementary file 7 — Comparison of selected phenotypic traits for the studied pig breeds. (DOCX 19 kb) [file 12863_2018_681_MOESM7_ESM.docx]

**Additional File 7**. Comparison of selected phenotypic traits for the studied pig breeds.

| Number of teats* | | | T-test P-value | | | | |
| --- | --- | --- | --- | --- | --- | --- | --- |
| Breed | Mean | sd | Breed | ZW | ZS | PUL |  |
| ZW | 14.09016 | 0.64287703 | ZW | x | 0.001256 | 0.17994 |  |
| ZS | 13.72826 | 0.97330592 | ZS |  | x | 0.000181 |  |
| PUL | 14.22523 | 0.88095697 | PUL |  |  | x |  |
| Average back-fat thickness^a^ | | | T-test P-value | | | | |
| Breed | Mean | sd | Breed | ZW | ZS | PL | PUL |
| ZW | 18.03357 | 4.64251942 | ZW | x | 1.17E-12 | 7.16E-55 | 2.4575E-30 |
| ZS | 23.134 | 3.53206764 | ZS |  | x | 1.97E-94 | 1.3217E-70 |
| PL | 9.267045 | 1.41272396 | PL |  |  | x | 8.096E-32 |
| PUL | 12.46737 | 2.47040878 | PUL |  |  |  | x |

*-no reproduction performance traits were available for PL breed

^a^ – Back-fat thickness measurements were made in animals aged 150-240 days using a Piglog 105 ultrasonic device in points:

P2 - back fat thickness behind the last rib, 3 cm from the midline of the ridge [mm]

P4 - back fat thickness behind the last rib, 8 cm from the midline of the ridge [mm]

In order to eliminate the differences resulting from the measurements of back-fat thickness of animals with different body weights, the results were standardized to 110 kg of body weight according to the formula:


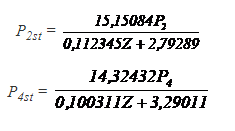


Z- body weight at the day of measurement

Than average back-fat thickness was presented as a mean of standardized P2 and P4 measurements.
